# Androgenesis in *indica* rice: A comparative competency in development of doubled haploids

**DOI:** 10.1371/journal.pone.0267442

**Published:** 2022-05-05

**Authors:** Byomkesh Dash, Sudhansu Sekhar Bhuyan, Sandeep Kumar Singh, Manjusha Chandravani, Nibedita Swain, Prachitara Rout, Jawahar Lal Katara, Parameswaran C., Devanna B. N., Sanghamitra Samantaray

**Affiliations:** 1 Crop Improvement Division, ICAR-National Rice Research Institute, Cuttack, Odisha, India; 2 Department of Plant Breeding and Genetics, College of Agriculture, Odisha University of Agriculture and Technology, Bhubaneswar, Odisha, India; United States Department of Agriculture, UNITED STATES

## Abstract

Rice is critical to global food security which demands immediate attention to meet the ever-growing population. Development of improved variety is the major focus area of research, in which doubled haploid (DH) technology plays a vital role. Since, androgenesis shows its potential in DH production, this method was not capitalized specially in *indica* rice due to due to its recalcitrant nature to tissue culture. Success of androgenesis is governed by many important factors such as stage of anther, pre-treatment conditions, accurate concentrations of media, and plant growth regulators. Though reports of androgenesis are abundant in rice, most of them either used *japonica* or a specific cultivar of *indica* rice ecotypes. In this study, a media combination was established which is successful in producing doubled haploids from F_1_s of Savitri x Pokkali, IR20 x Mahulata along with the popular *indica* hybrids of Arize 8433DT, Arize 6453, Arize Bold, and Swift Gold. Out of 12 different media combinations tested, and 5 different durations of cold-treatments studied, N6 media with 2,4-D (2.0 mg/l) and BAP (0.5 mg/l) with 7^th^ day cold pre-treatment was found to be most effective in all of the F_1_s for callus induction. Among all the F_1_s, rice hybrid, Arize 8433DT showed highest of 52% callus induction. In case of green shoot regeneration, MS media with NAA (0.5 mg/l), BAP (2.0 mg/l) and Kn (1.0 mg/l) (MS+C4) was found to be the most efficient of six treatments studied with highest of 58.25% regeneration in Arize 8433DT. Further, MS+C4 in combination with proline (5.0 mg/l) increased the regeneration rate to 85.99%. Besides, MS media with NAA (1.0 mg/l), Kn (0.1 mg/l) and 50 g/l sucrose was found to be most efficient for supporting root induction in all F1s. This study claims the establishment of genotype independent androgenic protocol for *indica* rice which could be capitalized in *indica* rice improvement.

## 1. Introduction

Rice is the predominant cereal grain for a substantial portion of the world’s population, particularly in Asia. It is estimated by 2050, the annual global demand for rice will be increased by 25% due to the increase of population. In Asia, food security has traditionally been defined as maintaining stable prices for rice in the major urban markets of a country [1] where it is the staple food of more than 50% of the population. Despite the fact that rice plays an important role in the food and agrarian ecosystems, traditional rice-producing countries are facing issues such as declining arable land owing to industrialization, resource constraints, and climate change consequences. As a result, defining strategies to safeguard the future of rice agriculture and its role in providing food security is crucial. In recent decades, rice productivity has increased dramatically as a result of the creation of high yielding varieties [2]. However, with the frequent occurrence of intense harsh/unfavorable climatic conditions including drought, flood, heat, salinity and heavy metal toxicity along with emerging pest and diseases, the yield is affected drastically, reducing the insufficient production [3]. Thus, more emphasis is given on development of climate resilient rice breeds with the input of less labor and limited time [4]. Though utilization of conventional and molecular breeding procedures could generate several high-yielding inbred and hybrid rice varieties till now [5], various changes in climatic environments, susceptibility to diseases and pests, exorbitant seed cost, chaffy or sterile grains has negatively affected the grain yield [6, 7]. To address these issues, it is necessary to make rapid changes in order to substantially cut breeding cycles and maintain the crop under unpredictably changing environmental conditions. Doubled Haploid (DH) technology have proven to be an effective tool for plant breeding, producing homozygous lines within a short span of time [8].

Anther culture is regarded as an advantageous technique in plant breeding to rapidly develop homozygous lines and augment selection efficiency. The foremost benefit of anther culture is regaining of genetically fixed doubled haploid (DH) plants. The efficacy of this technique is determined by the productivity of haploid plant regeneration from the anther derived microspores and subsequently conversion of these haploids to doubled haploids through genome doubling [9]. The duration for producing DH lines requires a single generation while the conventional breeding needs 7–8 generations of self-pollination to achieve the pure lines. Besides, DH attains 100% homozygosity whereas there is a chance 0.8% segregation in conventional breeding. Additionally, DH technology has shown its efficiency in contributing towards rapid cultivar development [10], generation of inbred parental lines [11], QTL/gene mapping [12], acquiring novel gene of interest from wild rice varieties. The potentiality in stabilizing transgenic lines [13] and reducing undesirable events of hemizygosity during transgenesis and genome editing has also been established [14]. The easy aggregation of recessive gene in homozygous condition within a single generation allows the expression of recessive traits like eui (elongated uppermost internode) genes [15], xa13 (bacterial blight resistance) [16]. Doubled haploidy approach coupled with conventional breeding and other biotechnological approaches led to development of a number of rice varieties for pest and disease resistance, high yield and good quality grains [17].

Androgenesis has been utilized to develop a number of rice varieties and improved breeding lines mostly in *japonica* cultivars [18]. However, *indica* cultivars was found to be recalcitrant to tissue culture including anther culture which hinders the success in rice improvement [19, 20]. Anther culture is of two steps: the first step involves the induction of calli from microspores and the next step deals with the regeneration of green plants from the calli [21]. However, there are a number of key factors which affect the success of anther culture such as the maturity of the donor plant [22], genotypic variation [23], microspore developmental stages [24], panicle pre-treatment [25], culture media [26] and growth conditions [27]. Besides, there are other problems such as anther necrosis, poor androgenesis frequencies and albino plant regeneration which have been blocking the successful application of anther culture technique(s) in *indica* rice breeding [28]. Changes in hormone composition [29], carbohydrate source [30], osmotic stress [31] have also been used to improve the regenerability of rice callus cultures. A two-step anther culture process involving various physical and chemical factors is found to be successful in *indica* rice [32]. However, this technique in anther culture could not be capitalized due to low rate of callusing response, frequent albino shoot regeneration, anther necrosis and genotype dependency. Therefore, the present study was taken up to establish a genotype independent androgenic method for *indica* rice by manipulating physical and chemical factors associated with anther culture.

## 2. Materials and methods

### 2.1 Plant material

A total of 6 *indica* genotypes were used in this study: the F_1_seeds of 4 popular *indica* rice hybrids, such as Arize 8433DT, Arize 6453, Arize Bold, Swift Gold were obtained from M/S Bayer, Hyderabad, India and 2 F_1_s of IR20 x Mahulata, and Savitri x Pokkali developed at ICAR-NRRI, Cuttack. All the seeds were sown in seed-bed for seedling purpose. The seedlings of 26–30 days old (approx. 3–4 leaf stage) were transplanted in main field with proper spacing of 20 x 15 cm. The recommended dose of fertilizer of 120-50-50 (kg/ha) of N-P-K was applied. The nitrogen (N) was applied in three split doses as per the package and practices of the crop and need based protection measures were taken.

### 2.2 Explants collection and pre-treatment

Well-developed and healthy boots from both primary and secondary tillers were collected at early morning (6am– 7am) from the field. Each boot was wiped cleaned with 70% ethanol and wrapped in a non-absorbent wet cotton followed by enclosing in polythene bag to prevent moisture loss and to maintain the pollen viability. Flag leaf and first leaf were trimmed from the boots keeping the penultimate sheath and node intact before the pre-treatment. A brief period of low temperature (10˚C) for 2–8 days at dark condition was maintained as cold pretreatment for the processed boots. Proper microspore stage of anther culture was selected following the acetocarmine staining method where the boots of 14–18 cm length positioned between flag leaf and penultimate leaf was taken as reference for harvesting the boots as maximum number of microspores are in early to late uninucleate. Further, the boots were surface sterilized with 70% ethanol for 4 min followed by 4% NaOCl for 2 min and rinsed three times with sterile de-ionized water. Before culturing of anthers of F_1_s, the anthers with mid to late uninucleate stages of microspore that were determined by cytological analysis, (25–30 anthers of individual F_1_s) were uniformly cultured over the surface of the media.

### 2.3 Culture media and incubation condition

N6 [33] supplemented with 1.0–2.0 mg/l NAA, 1.0–2.0 mg/l 2,4-D, 0–0.5 mg/l BAP and 0–0.5 mg/l Kn (designation—C1 to C6) (**Table 1**) was formulated for callus induction; media pH 5.8 using 0.1N NaOH/HCl was standardized. The medium was supplemented with 30 gm maltose as sole carbon source and solidified with 0.7% (w/v) agar-agar. Normally, 25ml of molten media was distributed in to culture tubes (25x150mm), plugged with non-absorbent cotton and sterilized at 121°C (15psi) for 15min. After sterilization the tubes were slanted (45°) for increased surface area of contact for culturing anthers.

**Table 1:** Callus induction (%) against days of pre-treatment.

Sl. No.	Genotype	Combination	N6 based media Combination					Days of pre-treatment*			
			NAA	2,4-D	BAP	Kn	2^nd-d^	4^th-c^	5^th-b^	7^th-a^	8^th-a^
1	Arize 8433DT^a^	C1^d^	2.0	-	-	-	0	0	0	0	0
		C2^b^	2.0	-	-	0.5	20	26	30	46	29
		C3^b^	1.0	1.0		0.5	23.5	28	31.16	47.65	38.6
		C4^c^	-	2.0	-	-	6	9	11	14.2	16
		C5^a^	-	2.0	0.5	-	26	31.2	37.55	52	45
		C6^b^	1.0	1.0	0.5	-	22	28	33	34.67	39.54
2	Arize 6453^b^	C1^d^	2.0	-	-	-	0	0	0	0	0
		C2^b^	2.0	-	-	0.5	20.12	22	28	35.29	38
		C3^b^	1.0	1.0		0.5	12.35	15.14	20	35.56	31.2
		C4^c^	-	2.0	-	-	6.17	7.56	8.14	12	7.58
		C5^a^	-	2.0	0.5	-	17.5	26	29.6	40	36.25
		C6^b^	1.0	1.0	0.5	-	13.25	16.21	22.13	36	29.14
3	ArizeBold^d^	C1^d^	2.0	-	-	-	0	0	0	18.47	11.14
		C2^b^	2.0	-	-	0.5	3	4.12	9.14	0	0
		C3^b^	1.0	1.0		0.5	2	4.12	6.45	16.78	9.15
		C4^c^	-	2.0	-	-	2.16	3.18	6.35	11.35	9.39
		C5^a^	-	2.0	0.5	-	0	5.5	11.35	23.6	17
		C6^b^	1.0	1.0	0.5	-	4.18	4.39	9.38	14.38	10.38
4	Swift Gold^d^	C1^d^	2.0	-	-	-	0	0	0	0	0
		C2^b^	2.0	-	-	0.5	0	3.98	9.37	17.38	11.36
		C3^b^	1.0	1.0		0.5	2.87	2.35	3.95	12.35	9.35
		C4^c^	-	2.0	-	-	2.35	1.39	3.59	8.37	6.27
		C5^a^	-	2.0	0.5	-	0	6.15	12.4	25.3	19
		C6^b^	1.0	1.0	0.5	-	3.65	2.58	3.25	10.25	6.98
5	IR20 x Mahulata^b^	C1^d^	2.0	-	-	-	0	0	0	0	0
		C2^b^	2.0	-	-	0.5	7.14	11.35	21.36	22.36	26.35
		C3^b^	1.0	1.0		0.5	8.79	14.35	22.35	23.65	27.35
		C4^c^	-	2.0	-	-	5.24	7.35	9.35	11.27	17.35
		C5^a^	-	2.0	0.5	-	15.2	23.85	31	40.2	46
		C6^b^	1.0	1.0	0.5	-	13.58	17.35	26.37	34.28	40.11
6	Savitri x Pokkali^c^	C1^d^	2.0	-	-	-	0	0	0	0	0
		C2^b^	2.0	-	-	0.5	2.37	3.85	16.97	24.33	20.14
		C3^b^	1.0	1.0		0.5	3.58	5.34	9.87	21.54	18.35
		C4^c^	-	2.0	-	-	2.54	6.87	8.35	10.35	9.31
		C5^a^	-	2.0	0.5	-	0	13.25	20.6	34	28.55
		C6^b^	1.0	1.0	0.5	-	3.47	11.24	14.78	28.98	21.35

Means sharing the same letter in a column were not significantly different in Duncan’s multiple comparison range test (p < 0.05).

* 10 replicates per treatment; repeated twice.

The anthers after culture were kept in total darkness at 25±2˚C till the emergence of callus from anthers. Microspore induced calli after attaining an average size of 2–3 mm were transferred to regeneration medium for green shoot regeneration.

Callus regeneration media was formulated using MS and N6 basal media with varied concentrations of NAA 0–0.5 mg/l, 2,4-D 0–0.5 mg/l, BAP 1.0–2.0 mg/l and Kn 1.0–2.0 mg/l (designation–C1 to C6) (**Table 2**). Secondly, to test the effect of proline on shoot regeneration, MS with 0.5 mg/l NAA, 2.0 mg/l BAP, 1.0 mg/l Kn and sucrose (30 gm/l) as only carbon source was fortified with 0–10.0 mg/l Proline (**Table 3**).

**Table 2:** Regeneration (%) against basal media type for each days of pre-treatment.

Sl. No	Genotype	PGR Combination				Combination	N6^b^ with Days of pre-treatment*					MS^a^ with Days of pre-treatment*				
		NAA	2,4-D	BAP	Kn		2^nd-c^	4^th-bc^	5^th-abc^	7^th-a^	8^th-ab^	2^nd-c^	4^th-bc^	5^th-abc^	7^th-a^	8^th-ab^
1	Arize 8433DT^a^	-	-	1.0	2.0	C1^d^	0	0	0	0	0	0	2.5	3.9	5.62	4.07
		-	-	2.0	1.0	C2^d^	0	0	0	0	0	2.4	3.2	3.87	6.27	5.87
		0.5	-	1.0	2.0	C3^b^	0	0.09	0.15	0.35	0.21	32.8	35.89	43.57	46.7	41.5
		0.5	-	2.0	1.0	C4^a^	0	0.11	0.24	0.5	0.39	40.27	45.87	49.25	58.25	53.67
		-	0.5	1.0	2.0	C5^c^	0	0	0	0	0	15.24	18.52	20.58	27.22	21.35
		-	0.5	2.0	1.0	C6^c^	0	0	0	0	0	17	21.56	24.66	29.5	26.55
2	Arize 6453^a^	-	-	1.0	2.0	C1^d^	0	0	0	0	0	0	2.56	3.68	5.7	4.26
		-	-	2.0	1.0	C2^d^	0	0	0	0	0	0	3.82	5.24	9.43	6
		0.5	-	1.0	2.0	C3^b^	0	0	0.12	0.29	0.17	25.08	26.39	28.54	35.21	32.05
		0.5	-	2.0	1.0	C4^a^	0	0.08	0.22	0.47	0.36	38.22	40.35	44	47.16	45.25
		-	0.5	1.0	2.0	C5^c^	0	0	0	0	0	17.55	20.8	23.5	26.3	24.52
		-	0.5	2.0	1.0	C6^c^	0	0	0	0	0	20.57	22.8	24.45	29.21	26.4
3	Arize Bold^a^	-	-	1.0	2.0	C1^d^	0	0	0	0	0	0	3.51	5.27	8	6.23
		-	-	2.0	1.0	C2^d^	0	0	0	0	0	2.9	4.52	8.23	11.5	9.54
		0.5	-	1.0	2.0	C3^b^	0	0.04	0.1	0.25	0.19	23.46	26.22	29.42	36.28	33.7
		0.5	-	2.0	1.0	C4^a^	0.12	0.23	0.35	0.68	0.51	33.4	35.8	39.65	44.38	42.5
		-	0.5	1.0	2.0	C5^c^	0	0	0	0	0	16.28	17.65	19.9	23.73	20.54
		-	0.5	2.0	1.0	C6^c^	0	0	0	0	0	19.6	21	22.9	26.41	24.7
4	Swift Gold^b^	-	-	1.0	2.0	C1^d^	0	0	0	0	0	0	0.99	3.54	5.22	4
		-	-	2.0	1.0	C2^d^	0	0	0	0	0	0	1.25	3	6.81	5.22
		0.5	-	1.0	2.0	C3^b^	0	0	0.02	0.1	0.07	18.55	22.34	24.7	29.81	26.47
		0.5	-	2.0	1.0	C4^a^	0	0.06	0.14	0.37	0.26	25.41	29.44	32.48	36.42	33.2
		-	0.5	1.0	2.0	C5^c^	0	0	0	0	0	8.3	10.58	13.9	17.34	15.5
		-	0.5	2.0	1.0	C6^c^	0	0	0	0	0	8.9	11.32	12.56	15.28	14.3
5	IR20 x Mahulata^a^	-	-	1.0	2.0	C1^d^	0	0	0	0	0	3.55	5.84	6.24	9.25	6.44
		-	-	2.0	1.0	C2^d^	0	0	0	0	0	3.9	6	9.65	13.58	11.3
		0.5	-	1.0	2.0	C3^b^	0.07	0.2	0.28	0.61	0.49	26.3	30.75	32.5	39.21	35.6
		0.5	-	2.0	1.0	C4^a^	0.1	0.3	0.52	0.89	0.65	40.23	41.65	44	46.84	49.42
		-	0.5	1.0	2.0	C5^c^	0	0	0	0	0	16.31	19	21.87	25.41	23.3
		-	0.5	2.0	1.0	C6^c^	0	0	0	0	0	19.34	21.34	26.7	30.4	27.95
6	Savitri x Pokkali^c^	-	-	1.0	2.0	C1^d^	0	0	0	0	0	0	2.58	4	7.29	6.27
		-	-	2.0	1.0	C2^d^	0	0	0	0	0	0	0.8	2.57	5.8	3.22
		0.5	-	1.0	2.0	C3^b^	0	0	0.07	0.18	0.13	5.23	7	10.25	15.21	12.4
		0.5	-	2.0	1.0	C4^a^	0	0.05	0.09	0.25	0.14	10.27	12.65	17.5	24.97	21
		-	0.5	1.0	2.0	C5^c^	0	0	0	0	0	3.55	5.23	7	10.25	8.6
		-	0.5	2.0	1.0	C6^c^	0	0	0	0	0	4	6.42	8.22	13.8	10.51

(**C1-C6**: Designates 6 different combinations of PGRs with N6 and MS basal media**);** Means sharing the same letter in a column were not significantly different in Duncan’s multiple comparison range test (p < 0.05).

* 10 replicates per treatment; repeated twice.

**Table 3:** Green shoot regeneration (%) against various concentrations of proline.

Sl No.	Genotype	MS + NAA (0.5 mg/l) + BAP (2.0 mg/l) + Kn (1.0 mg/l) + Sucrose (30 g/l)				Increase in regeneration(%)
		Proline (0 mg/l)^b^	Proline (1.0 mg/l)^c^	Proline (5.0 mg/l)^a^	Proline (10.0 mg/l)^d^	
1	Arize 8433DT	58.25b	24.97c	85.99a	11.09d	27.74
2	Arize 6453	47.16b	19.42c	83.21a	8.32d	36.05
3	Arize Bold	44.38b	21.77c	81.6a	8.32d	37.22
4	Swift Gold	36.42b	18.48c	76.26a	10.18d	39.84
5	IR20 x Mahulata	49.42b	11.1d	62.7a	16.64c	13.28
6	Savitri x Pokkali	24.97b	8.32d	61a	11.09c	36.03

Means sharing the same letter in a column were not significantly different in Duncan’s multiple comparison range test (p < 0.05).

* 10 replicates per treatment; repeated twice.

Thirdly, assessing the carbon source effect on shoot regeneration, varied concentrations of sucrose (15, 30, 60 gm/l) (designation S15, S30, S60) and maltose (15, 30, 60 gm/l) (designation M15, M30, M60) were taken in MS with 0.5 mg/l NAA, 2.00 mg/l BAP and 1.00 mg/l Kn (**Table 4**). The quantity of callus used and the incubation conditions maintained were similar to that of shoot regeneration. The pH of each and every combination of medium was adjusted to 5.8 using 0.1N NaOH/HCl and solidified with 0.7% (w/v) agar-agar. The medium was then dispensed into 25x150 mm tubes, plugged with cotton, and sterilized at 15 psi for 15 minutes.

**Table 4:** Shoot regeneration (%) against various carbon sources.

Sl No.	Genotype	MS + NAA (0.5 mg/l) + BAP (2.0 mg/l) + Kn (1.0 mg/l)		Combination	Callus response to shoot regeneration (%)*
		Sucrose (g/l)	Maltose (g/l)		
1	Arize 8433DT^a^	15	-	S15^c^	28.65
		30	-	S30^a^	58.25
		60	-	S60^d^	16.37
		-	15	M15^c^	24.36
		-	30	M30^b^	40.25
		-	60	M60^d^	13.68
2	Arize 6453^ab^	15	-	S15^c^	21.36
		30	-	S30^a^	47.16
		60	-	S60^d^	13.89
		-	15	M15^c^	23.5
		-	30	M30^b^	29.67
		-	60	M60^d^	11.89
3	ArizeBold^ab^	15	-	S15^c^	27.6
		30	-	S30^a^	44.38
		60	-	S60^d^	15
		-	15	M15^c^	18.34
		-	30	M30^b^	31.47
		-	60	M60^d^	14.58
4	Swift Gold^bc^	15	-	S15^c^	14.35
		30	-	S30^a^	36.42
		60	-	S60^d^	10.58
		-	15	M15^c^	18.97
		-	30	M30^b^	22.47
		-	60	M60^d^	14.78
5	IR20 x Mahulata^a^	15	-	S15^c^	31.47
		30	-	S30^a^	46.84
		60	-	S60^d^	21.45
		-	15	M15^c^	32.54
		-	30	M30^b^	33.87
		-	60	M60^d^	14.78
6	Savitri x Pokkali^c^	15	-	S15^c^	11.24
		30	-	S30^a^	24.97
		60	-	S60^d^	9.35
		-	15	M15^c^	13.4
		-	30	M30^b^	18.75
		-	60	M60^d^	6.97

Means sharing the same letter in a column were not significantly different in Duncan’s multiple comparison range test (p < 0.05).

* 10 replicates per treatment; repeated twice.

Each tube was inoculated with 4 calli only, in order to counter the osmotic stress exerted by sugar in the medium. Post inoculation the cultures were incubated under 3000 lux white fluorescent light with 16hr photoperiod. Relative humidity and temperature were maintained at 60–70% and 25±2˚C, respectively. This constant incubation conditions were maintained throughout the study.

### 2.4 Microshoots to rooting

The green regenerants (8–12 cm) with healthy vigor were transferred to MS supplemented with 1.0–2.0 mg/l NAA, 0.1–0.5 mg/l Kn along with 30–50 gm/l sucrose (designation R1 to R8) (**Table 5**) and solidified with 8% (w/v) agar-agar for root induction. The sterilized medium in each test tube was inoculated with 1 regenerant each, to allow proper growth and development implementing the standard incubation conditions.

**Table 5:** MS based rooting medium efficiency.

Sl No.	Genotype	MS + PGR (mg/l) + Carbon source (g/l)			Combination	Root induction* (%)
		NAA	Kn	Sucrose		
1	Arize 8433DT^a^	1.0	0.1	30.0	R1^d^	52
		2.0	0.1	30.0	R2^f^	35.12
		1.0	0.5	30.0	R3^g^	29.61
		2.0	0.5	30.0	R4^h^	24.8
		1.0	0.1	50.0	R5^a^	100
		2.0	0.1	50.0	R6^b^	78.13
		1.0	0.5	50.0	R7^c^	65
		2.0	0.5	50.0	R8^e^	47.25
2	Arize 6453^a^	1.0	0.1	30.0	R1^d^	63
		2.0	0.1	30.0	R2^f^	29.13
		1.0	0.5	30.0	R3^g^	32.14
		2.0	0.5	30.0	R4^h^	22.8
		1.0	0.1	50.0	R5^a^	100
		2.0	0.1	50.0	R6^b^	82.13
		1.0	0.5	50.0	R7^c^	55
		2.0	0.5	50.0	R8^e^	32.57
3	ArizeBold^a^	1.0	0.1	30.0	R1^d^	52
		2.0	0.1	30.0	R2^f^	35.12
		1.0	0.5	30.0	R3^g^	29.61
		2.0	0.5	30.0	R4^h^	24.8
		1.0	0.1	50.0	R5^a^	100
		2.0	0.1	50.0	R6^b^	78.13
		1.0	0.5	50.0	R7^c^	65
		2.0	0.5	50.0	R8^e^	47.25
4	Swift Gold^a^	1.0	0.1	30.0	R1^d^	49
		2.0	0.1	30.0	R2^f^	32.48
		1.0	0.5	30.0	R3^g^	26.18
		2.0	0.5	30.0	R4^h^	22.6
		1.0	0.1	50.0	R5^a^	100
		2.0	0.1	50.0	R6^b^	80.13
		1.0	0.5	50.0	R7^c^	64
		2.0	0.5	50.0	R8^e^	52.12
5	IR20 x Mahulata^a^	1.0	0.1	30.0	R1^d^	47.48
		2.0	0.1	30.0	R2^f^	35.12
		1.0	0.5	30.0	R3^g^	26.74
		2.0	0.5	30.0	R4^h^	22.92
		1.0	0.1	50.0	R5^a^	100
		2.0	0.1	50.0	R6^b^	69.42
		1.0	0.5	50.0	R7^c^	66
		2.0	0.5	50.0	R8^e^	42.88
6	Savitri x Pokkali^a^	1.0	0.1	30.0	R1^d^	58.06
		2.0	0.1	30.0	R2^f^	32.67
		1.0	0.5	30.0	R3^g^	29.61
		2.0	0.5	30.0	R4^h^	26.28
		1.0	0.1	50.0	R5^a^	100
		2.0	0.1	50.0	R6^b^	60.15
		1.0	0.5	50.0	R7^c^	49
		2.0	0.5	50.0	R8^e^	48.23

Means sharing the same letter in a column were not significantly different in Duncan’s multiple comparison range test (p < 0.05).

* 10 replicates per treatment; repeated twice.

### 2.5 Acclimatization and net-house transfer

Regenerants with well-formed roots were removed from the medium and thoroughly washed and kept in tap water under room temperature for 48 hrs. Post hardening regenerants were transplanted to 12” pots in net-house and necessary care were taken for proper growth and development. Recommended dose of N-P-K were applied and need based irrigation were given.

### 2.6 Ploidy evaluation

All the developed plantlets were assessed based on morphological characters: plant height, spikelet fertility (%) after reaching full maturity. Plants with normal vigor and appearance with average fertility could be classified as diploids/putative DHs, Polyploids were screened by their gigantic growth habits. Likewise, haploids were screened out for their short stature [29, 34] with infertile inflorescence.

### 2.7 Marker based assessment for true DHs

Identified diploids/putative DHs were subjected to marker-based assessment for discrimination of diploids (somatic tissue derived plants) from the assertive DHs. Cetyltrimethyl ammonium bromide (CTAB) method [35] was used to extract the total genomic DNA of the individual plants. PCR (polymerase Chain Reaction) based gene amplification was carried out using reaction mixture 10ul (**Table 6**). The reaction was carried under initial denaturation at 94˚C for 4 min followed by 35 cycles each of denaturation (94˚C; 30sec), annealing (56˚C; 45 sec) and elongation (72˚C; 1 min). The final step of the reaction was carried under 72˚C for 10min as final elongation that marks the completion of the polymerization reaction. After completion of the reaction the product was assayed against 100bp DNA ladder (O’Gene Ruler 100bp DNA ladder; MBI Ferment Inc., Maryland, USA) in 2.5% (m/v) Agarose (Bangalore Genei, Bangalore, India) gel made in 1x Tris-borate-ethylenediamine tetra acetic acid (TBE buffer). Ethidium bromide was used as dying agent and the bands were visualized under UV gel documentation system (Syngene, Cambridge, UK).

**Table 6:** PCR reaction mixture.

Sl. No.	Components	Concentration	Manufacturer	Volume (ul)
1	Template DNA	25ng	-	1.00
2	PCR buffer (1x)	1x	GCC biotech, India	1.00
3	Forward primer	10pM	Eurofins genomics, India	0.50
4	Reverse primer	10pM	Eurofins genomics, India	0.50
5	dNTPs	0.25mM	GCC biotech, India	1
6	MiliQ water	-	Eppendorf, India	5.80
7	Taq-pol.	5unit/ul	GCC biotech, India	0.20

### 2.8 Statistical analysis of different media responses

Each treatment of callus induction medium was inoculated with 10 cultures (each tube containing 40–50 anthers avg.) of each type. While the green shoot regeneration treatments were tested with 40 calli (each tube containing 4 calli; total 10 tubes each) for each type. The total culture setup was repeated twice and frequency of callus induction followed by green shoot regeneration was recorded. A comparative analysis between control, basal medium N6/MS only, and different treatments were assessed based upon completely randomized design (CRD). The callus response and shoot regeneration response were calculated according to the following equations:

$$Callus\;induction\;(\%)=\frac{no.of\;anthers\;producing\;callus}{total\;no.of\;anthers\;cultured}\times 100$$

$$Callus\;regenartion\;(\%)=\frac{no.of\;callus\;producing\;green\;shoots}{total\;no.of\;callus\;cultured}\times 100$$

Responses of all the parameters (Callus induction in all genotypes w.r.t days of pre-treatment, variable concentration of plant growth regulators (PGR); Green shoot regeneration in all genotypes w.r.t days of pre-treatment, basal media type, variable concentration of PGR; Green shoot regeneration in all genotypes w.r.t variable proline concentrations; Green shoot regeneration in all genotypes w.r.t different concentrations of carbon sources; Root induction w.r.t variable concentration of PGR) were subjected to analysis of variance (ANOVA) using R software version 4.0.2. [36] and categorized according to Duncans’ multiple range test (DMRT) using the Web Based Agricultural Statistics Software Package (WASP 1.0, ICAR-CCARI) [37].

## 3. Results

### 3.1 Identification of correct booting stage for androgenesis

**Fig 1:**
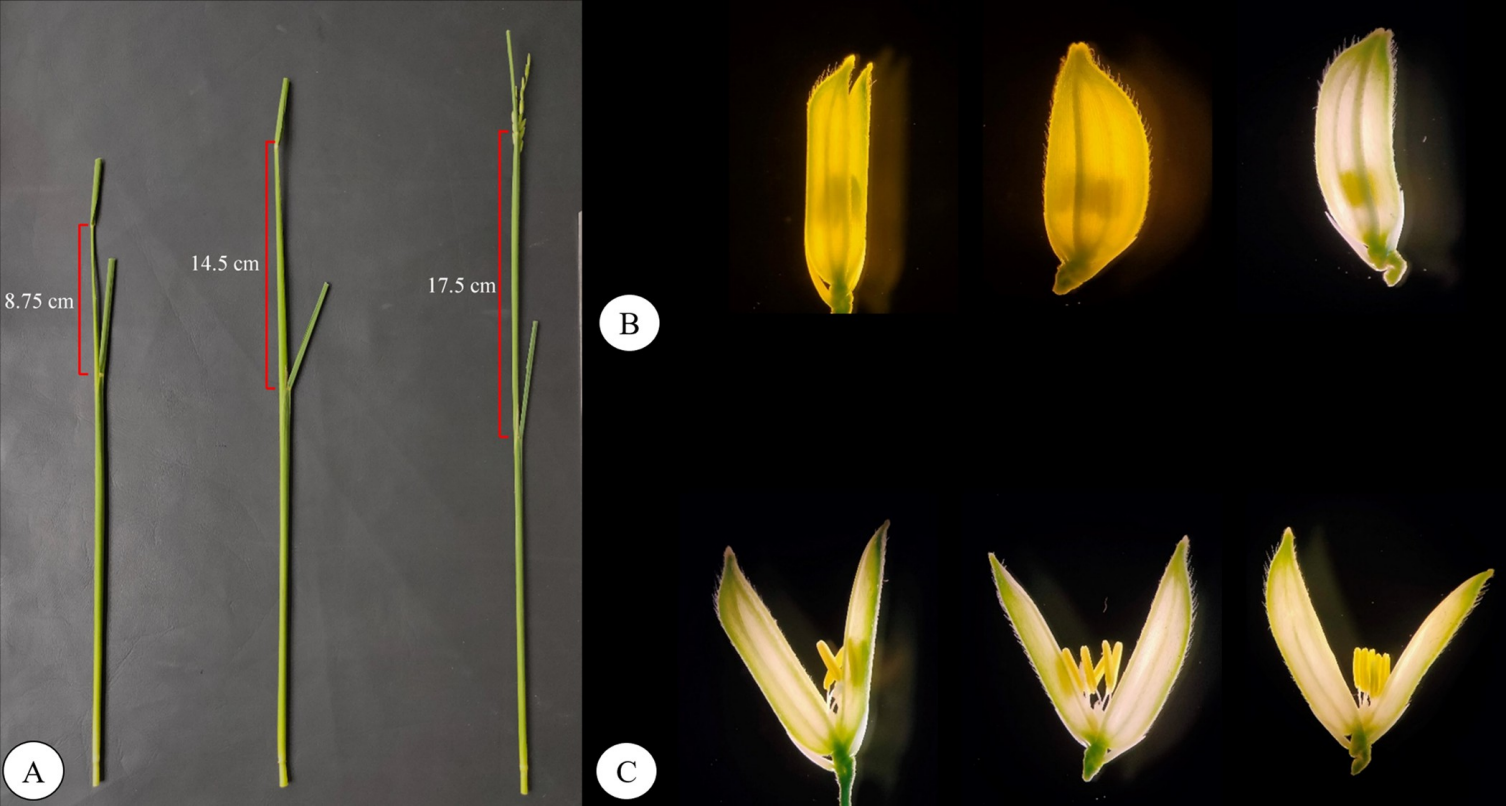
**A)** Boot showing flag leaf and penultimate leaf distance (the best one is 14–18 cm length) **B)** Difference in anther position of three different boots (closed flower) **C)** Difference in anther position of three different boots (opened flower).

Cytological analysis of the anthers revealed the boots possessing flag leaf and penultimate leaf distance in the range of 14–18 cm, have maximum number of microspores in early- to mid- uninucleate stage (**Fig 1**). More accurately, the anthers when positioned exactly in the middle of the floret, the microspores are mostly in early- to mid- uninucleate stage. As panicle contains many florets at different developmental stage, observation showed that the anthers above the mid-level in the floret have majority of bi-nucleate microspores. When most of the florets reach this stage, the panicle is partially exerted from the boots and becomes mostly unresponsive to androgenesis. On contrary, when the boots are too immature the anthers in the florets are close to the gynoecium or below the mid-level of the floret most of the microspores are at tetrad stage or near to uninucleate stage, which are both not useful for androgenesis. Hence, the correct stage for androgenesis is correlated to the early to mid-uninucleate that could be found mostly in the boots having avg. 14–18 cm distance between flag leaf and penultimate (first) leaf.

### 3.2 Pre-treatment of boots and callus induction

**Fig 2:**
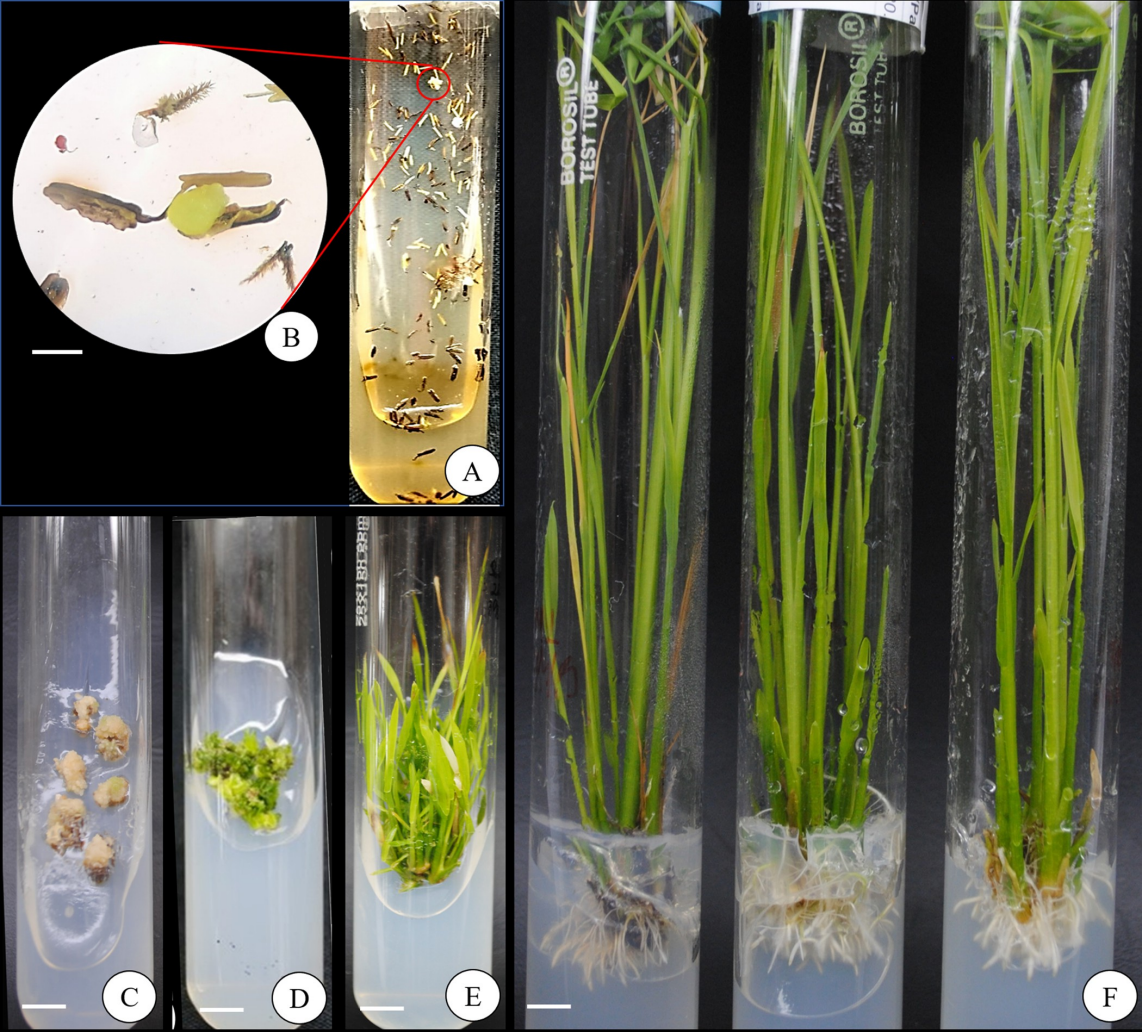
**A)** Callus induction in Arize 8433DT after 4 weeks of culture **B)** Enlarged view of the induced calli **C)** Calli of Arize 8433DT grown in MS + C4 after 2 weeks of culture **D)** Shoot initiation of Arize 6453 in MS + C4 after 2 weeks of culture **E)** Shoot enlargement of Arize 6453 in MS + C4 after 4 weeks of culture **F)** Root induction in regenerants after 2 weeks of culture in MS + R5 of IR20 x Mahulata.

The collected boots at the correct stage were incubated at 10˚C for different time periods. Initially, the anthers turned brown and then black after 3–6 weeks of culture. Subsequently, the anthers swelled and burst opened yielding small off-white globular perturbance on the surface (**Fig 2**). These small outgrowths later developed into calli. Most of the calli had off-white appearance with few exceptions of moist appearance. The appearance of moist calli increased upon lowering the incubation period of pre-treatment. Alternatively, increase of the pre-treatment period more than 8^th^ day resulted in reduction of overall callus induction. Out of 2–8 days of pre-treated boots, the boots at 7^th^ and 8^th^ day of treatment were found to be more responsive to the callus induction in N6 based medium fortified with 2.0 mg/l 2,4-D, 0.5 mg/l BAP (C5) and 30 g/l maltose (**Table 1**). The results obtained by subjecting the observations to ANOVA were also in accordance to the observed media combinations (**Table 7**). On the basis of the performance by various genotypes Arize 8433DT had the highest callus induction (52%) followed by IR20 x Mahulata (46%) and Arize 6453 (40%) while Swift Gold (25.3%) and Arize Bold (23.6%) were the least responsive. Similarly, among the various PGR combinations, C5 (2.0 mg/l 2,4-D, 0.5 mg/l BAP) was the most effective combination which produces highest callus induction in all the six tested genotypes followed by C6, C2 and C3, in decreasing order, while C4 and C1 were found to be the least responsive. In case of pre-treatment days, 7th day pre-treatment was found to be most responsive in all the genotypes tested except IR20 x Mahulata which had the best callus response of 46% under 8^th^ day of pre-treatment.

**Table 7:** ANOVA results for callus induction.

Sl. No.	Variables	Df	Sum Sq	Mean Sq	F value	Pr (>F)
1	Variety	5	24216	4843	123.11	<2e-16***
2	Combination	5	32972	6594	167.62	<2e-16***
3	Treatment	4	13947	3487	88.63	<2e-16***
4	Residuals	525	20654	39		

Signif. codes: 0 ‘***’ 0.001 ‘**’ 0.01 ‘*’ 0.05 ‘.’ 0.1 ‘ ‘ 1.

### 3.3 Effects of various basal media and pre-incubation conditions on green shoot regeneration

The N6 induced calli of 2–8 days of pre-treatment showing 3–4 mm growth size were transferred to MS and N6 based regeneration media fortified with 0–0.5 mg/l NAA, 0–0.5 mg/l 2,4-D, 1.0–2.0 mg/l BAP and 1.0–2.0 mg/l Kn. After 2 weeks of culture, all the MS based combinations showed increase in the callus size, ranging from 8–10 mm. However, a specific media type, MS combined with C4 (0.5 mg/l NAA, 2.0 mg/l BAP and 1.0 mg/l Kn) started showing green spots on the callus surface derived from 7^th^ and 8^th^ day pre-incubated boots. While other pre-treatments inoculated in the same MS+C4 based combination remained off-white. On contrary, there was no significant response observed in shoot regeneration in N6 based media combinations. Though N6+C4 (N6 supplemented with 2.00 mg/l BAP, 1.00 mg/l Kn and 0.50 mg/l NAA) showed little enlargement in size of the calli, other N6 combinations (**Table 2**) viz. C1-C6 did not show any response rather the calli turned brown in color for all pre-treatments. After 4 weeks of culture, the anthers derived from 7^th^ day pre-treatment produced 2–3 cm shoots in MS+C4 (MS + 0.5 mg/l NAA, 2.0 mg/l BAP and 1.0 mg/l Kn) while 1–2 cm shoots were observed in 8^th^ day pre-treated anthers derived calli indicating slow growth rate. The four-way ANOVA approach suggested role played by all four variables such as genotype, PGR combination, basal media type and days of pre-treatment are significant (p<0.001) with respect to the rate of green shoot regeneration (**Table 8**). Four genotypes, Arize 8433DT (58.25%), IR20 x Mahulata (49.42%), Arize 6453 (47.16%) and Arize Bold (44.38%) showed regeneration rate which were in close range to each other while only two, Swift Gold (36.42%) and Savitri x Pokkali (24.97%) genotypes had regeneration rates which were not in the same range. The observation suggested that regeneration is not affected by the genetic variability which is a remarkable accomplishment of this study. Among the various combinations of PGRs used, the combination C4 (0.5 mg/l NAA, 2.0 mg/l BAP and 1.0 mg/l Kn) was found to be most responsive among the 6 combinations tested, followed by C3, C6, C5 and C2, C1 as least responsive. For the two types of basal media tested for regeneration, MS was found to be most responsive while N6 being least responsive. In case of days of pre-treatment correlated to the rate of green shoot regeneration, 7^th^ day pre-treated anthers derived calli have the highest rate of green shoot regeneration in all the genotypes with the exception of IR20 x Mahulata, which had its highest response under 8^th^ day of pre-treatment. Moreover, the watery like calli only grew in size, never showing any green spots in any of the media combinations tested.

**Table 8:** ANOVA results for effects of various basal media and pre-incubation conditions on green shoot regeneration.

Sl No.	Variables	Df	Sum Sq	Mean Sq	F value	Pr(>F)
1	Variety	5	8223	1645	30.08	<2e-16***
2	Combination	5	38118	7624	139.44	<2e-16***
3	Media	1	92343	92343	1688.94	<2e-16***
4	Treatment	4	2865	716	13.10	2.02e-10***
5	Residuals	1064	58174	55		

Signif. codes: 0 ‘***’ 0.001 ‘**’ 0.01 ‘*’ 0.05 ‘.’ 0.1 ‘ ‘ 1.

### 3.4 Effect of proline on green shoot regeneration

The effect of proline on green shoot regeneration was studied using MS+C4 media combinations with sucrose 30 gm/l fortified with proline (0–10.0 mg/l). The two-way ANOVA marked the effect of both variables “Variety” (Genotypes) and “Combination” (Variable Proline Concentrations) as highly significant (p<0.001) (**Table 9**). All six genotypes showed similar response to the addition of proline suggesting that there is no significant difference among the genotypes for any given concentration of the proline in the medium making the use of proline independent of genotypic effect, often observed in *indica* genotypes for androgenesis. Among the 4 different concentrations of proline tested, 5.0 mg/l proline was found to be the most effective concentration for green shoot regeneration which increased the green shoot regeneration frequency by 31.69% (avg.) suggesting an alleged role of polyamines in green shoot regeneration. Here also, the 7^th^ day (except IR20 x Mahulata: 8^th^ day) pre-treated induced calli were cultured in proline supplemented media which showed tremendous increase in the green shoot regeneration as compared to control (MS + C4 + sucrose 30 gm/l + Proline 0 mg/l). A highest increase of 39.84% in green shoot regeneration in Swift Gold (76.26%) was observed over its control (36.42%), which was almost double as compared to its control. It was closely followed by Arize Bold (81.6%) showing 37.22% increment over its control (44.38%). Among the two crosses used Savitri x Pokkali and IR20 x Mahulata, former showed increment of 36.03% over its control while the later showed only 13.28% increase. Also, the increase of 13.28% is the least (**Table 3**) among all the genotypes under investigation.

**Table 9:** ANOVA results for effect of proline on green shoot regeneration.

Sl No.	Variables	Df	Sum Sq	MeanSq	F value	Pr (>F)
1	Variety	5	2347	469	13.57	5.5e-09***
2	Combination	3	46084	15361	444.04	<2e-16***
3	Residuals	63	2179	35		

Signif. codes: 0 ‘***’ 0.001 ‘**’ 0.01 ‘*’ 0.05 ‘.’ 0.1 ‘ ‘ 1.

### 3.5 Effect of different carbon sources on green shoot regeneration

In the third stage of experiment for green shoot regeneration, two different carbon sources viz. sucrose and maltose were tested with different concentrations supplemented with MS+C4, the highest responding media along with the 7^th^ day (except IR20 x Mahulata: 8^th^ day) pre-treated calli. The calli transferred to MS+C4 with 30 gm/l sucrose showed highest green shoot regeneration of 58.25% in Arize 8433DT followed by Arize 6453 (47.16%) and IR20 x Mahulata (46.84%), while Savitri x Pokkali (24.97%) was least responsive (**Table 4**); no green shoots were observed in MS+C4 supplemented with maltose (30 gm/l). Two-way ANOVA was also used for comparing the effect of type of sugar and its concentration used across all the genotypes which suggested both the genotype and sugar played significant role individually for determining the rate of green shoot regeneration (**Table 10**).

**Table 10:** ANOVA results for effect of different carbon sources on green shoot regeneration.

Sl. No.	Variable	Df	Sum Sq	Mean Sq	F value	Pr(>F)
1	Variety	5	3530	706.0	47.68	<2e-16***
2	Combination	5	11065	2213.1	149.47	<2e-16***
3	Residuals	97	1436	14.8		

Signif. codes: 0 ‘***’ 0.001 ‘**’ 0.01 ‘*’ 0.05 ‘.’ 0.1 ‘ ‘ 1.

### 3.6 Root induction, acclimatization and net-house transfer

**Fig 3:**
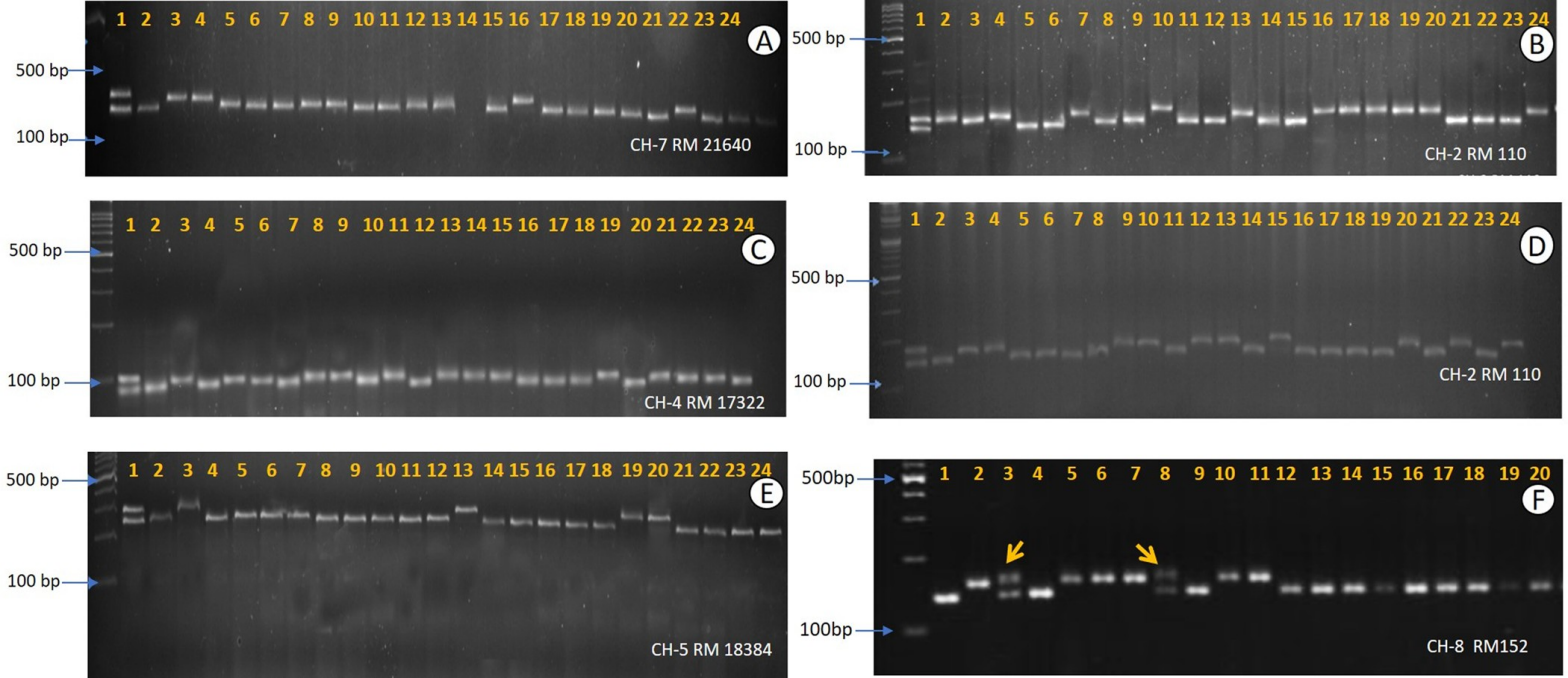
Discrimination of doubled haploid and heterozygous diploids using SSR markers. **A)** 1: Arize 8433DT; 2–24: Derivative DHs **B)** 1: Arize 6453; 2–24: Derivative DHs **C)** 1: Arize Bold; 2–24: Derivative DHs **D)** 1: Swift Gold; 2–24: Derivative DHs **E)** 1: F_1_s of Savitri x Pokkali; 2–24: Derivative DHs **F)** 1: IR20 2: Mahulata; 4–7 & 9–20: Derivative DHs; 3 & 8: Heterozygous diploids (arrows).

The regenerants generated from different treatments, of vigor size 8–10 cm were transferred to MS fortified with 1.0–2.0 mg/l NAA, 0.1–0.5 mg/l Kn and 30–50 gm/l sucrose and solidified with 8% (w/v) agar-agar. After 2 weeks of incubation under standard incubation conditions, 100% root induction was observed in MS fortified with 1.0 mg/l NAA, 0.1 mg/l Kn and 50 gm/l sucrose (R5) for all genotypes (**Table 5**). Two-way ANOVA for root induction frequency using the variables Genotype and Combination of PGR and sugar suggested the concentration of various PGRs along with sugar plays significant role as compared to the genotype (**Table 11**) suggesting the independency of genotype over root induction. After proper development of roots (root length > 5 cm) (**Fig 3**), the regenerants were thoroughly washed under tap water for removal of agar. Regenerants with healthy roots were acclimatized and transferred to the net house in batches, demonstrating 100% survival. Variations in terms of plant height, panicle size and grain form were observed in all the green plants.

**Table 11:** ANOVA results for root induction.

Sl No.	Variables	Df	Sum Sq	Mean Sq	F value	PR(>F)
1	Variety	5	268	54	2.316	0.0472*
2	Combination	7	81498	11642	503.871	<2e-16***
3	Residuals	131	3027	23		

Signif. codes: 0 ‘***’ 0.001 ‘**’ 0.01 ‘*’ 0.05 ‘.’ 0.1 ‘ ‘ 1.

### 3.7 Ploidy evaluation and marker-based assessment for true DHs

Developed regenerants were evaluated for their ploidy status based on their morpho-agronomic characters. Surprisingly, all the anther cultured (AC) derived plants were found to be true DHs (**Fig 3**) except for the IR20 x Mahulata derived plantlets through AC. Again, the IR20 x Mahulata based AC derived plantlets showed a varied degree of ploidy level. The height based morphological traits identified 20.1% haploids and 79.9% diploids/putative DHs in IR20 x Mahulata. Further, STMS marker-based assessment (**Fig 3**) showed only two plants as diploids showing heterozygous loci while rests were true DHs.

## 4. Discussion

DH used as speed breeding tool has paved a new way in improvement of rice crops. Though a number of rice varieties of *japonica* were developed through DH technology, it has not been capitalized in *indica* rice due to the major constraints like genotype dependency to media and albino shoot regeneration. Therefore, clearing the bottleneck of DH technology in view of improving the *indica* rice varieties has been in focus for recent years. Despite the fact that a number of androgenesis methods have been developed for *indica* rice [38], it was not able to produce appreciable quantity of DHs. Thus, this study focuses on manipulation of physical and chemical factors to develop an efficient method for production of large quantity of DHs. Moreover, effectiveness of pre-treatment duration and stages of microspore maturity was tested in 6 genotypes showing variable responses. However, different stages of microspores found in the boots, early- to late- uni-nucleate stage of microspores are the most responsive factors for efficient calli induction [29]. As the development of a boot is dependent upon the growth of a rice plant, determining the accurate stage of the microspore is difficult. Moreover, the position of anthers in an un-opened floret could be a better physical indicator depicting the microspore stage more accurately. Also, this trait could be physically linked to the leaf distance of the developing boots in the plants, making the identification easier [39].

Temperature (pre-treatment) is one of the most critical elements influencing callus induction [40]. However, the duration of pre-treatment varies within a species or from genotype to genotype [41]. Cold pre-treatment has been shown to reduce the deterioration of anther tissue and shield microspores from the toxicity of decaying anthers in a variety of species [42] ensuring the higher survivability of microspores in embryonic stage [43]. Cold pre-treatment enhances the callus induction by arresting the microspores in gametophytic stage and pushing them towards the sporophytic stage [44]. Regardless of the media tested, our finding also demonstrated that cold pre-treatment of spike had a beneficial effect on callus induction frequency. The study indicated that the spikes pre-treated for 7 and 8 days at 10˚C had the highest responsiveness for callus induction and subsequently for green shoot regeneration. The spikes of most of the genotypes showed highest callus induction with 7 days of pre-treatment, while IR20 x Mahulata showed best responsiveness for 8 days of pre-treatment. The reason might be the overall increase in the free amino acids including heat shock protein content of anthers during the cold pre-treatment that may be conducive to microspore adaptability to metabolic alterations and callus induction and protects the microspores from the chilling stress [45, 46]. Also, there was a report that prolonging the pre-treatment period often leads to a single base pair mutation (C to T creating missense mutation of Thr to Ile) causing infuriating issue of albinism in *indica* rice androgenesis [47]. The study also indicated the reduction of pre-treatment duration viz. less than 4 days or increasing the duration more than 8 days had negative influence on the callus induction. It may be caused due to the prolonged starvation of the microspores disconnected from the tapetum which rendered the microspores dead or necrotic [48]. The spikes treated for 2–4 days of cold shock formed calli with watery texture which did not respond to shoot regeneration.

Accompanying the temperature and duration of cold pre-treatment, the success of androgenesis is also dependent upon the constituent of commonly used basal media like MS, N6 of which N6 was found to be more responsive in development of calli whereas MS was found efficient in green shoot regeneration which is corroborated to the findings of Rout et al. [26]. Though use of 2,4-D alone in the medium induces callusing in *indica* x *japonica* [49], the combination of 2,4-D (2.0 mg/l) and BAP (0.5 mg/l) in N6 media enhanced callus induction in *indica* genotypes. Replacing BAP with Kn drastically reduces the response of callus induction [50]. The observations made from the study suggested an alleged synergistic role of both auxin and cytokinin in callus induction. Besides, the beneficial effect of maltose was proved in callus induction [51, 52]. This might be due to the slow hydrolysis of maltose yielding only glucose, to support the microspore’s response into calli whereas fructose released from the hydrolyzed sucrose may inhibit the callus development by plasmolyzing the microspores [29].

Apart from callus induction, green shoot regeneration is the most important stage of androgenesis without which the whole experiment is obsolete. Often the success of androgenesis is assessed in terms of green shoot regeneration frequency. Various factors like type of basal media, PGRs concentrations and combinations, condition of anthers and genotype are found to influence not only the callus induction but also the green shoot regeneration. To increase the androgenic efficiency all these factors must be considered parallally during callus induction and green shoot regeneration. Of different combinations tested for PGRs with two different basal media (N6 and MS), MS based media showed significant response in terms of green shoot regeneration, which might be due to the presence of high concentrations of inorganic nitrogen in MS as compared to N6. This study is corroborated with Rout et al. [26]; Naik et al. [29]; Pattnaik et al. [39] showing the efficiency of MS media over other basal media combinations like SK1 and N6 in *indica* rice hybrids (CRHR32, BS6444G and 27P63).

Choosing a reliable carbon source along with correct basal media composition guides the regeneration of callus towards the success of androgenesis. Carbon and nitrogen are two most important macro nutrients of the medium. Carbon plays an important role in tissue culture media which compensate the energy requirement of proliferating cells. Sugars also regulate the osmotic pressure in the culture media. Sucrose and maltose are the two most widely used sugars in the culture media. Of the two sugars tested, sucrose showed its efficiency in production of green shoot regeneration at 30 gm/l. Increasing the concentration beyond 30 gm/l drastically reduced green shoot regeneration frequency. This might be due to the decrease of osmotic potential in the medium which restricts the absorption of nutrition. Similar result was reported in hybrid rice androgenesis recently [39].

Vitamins and amino acids are the preferential organic nitrogen source in the culture media. Under stress condition plants tends to accumulate several metabolites and amino acids specially proline, which is a common strategy for the protection and survival of cells under such conditions including callus induction, green shoot regeneration and embryogenesis [53–55]. This study showed increment of green shoot regeneration in proline fortified media for all tested genotypes except IR20 X Mahulata. This could be correlated to the drought tolerance of Mahulata [56] which enabled its F1s to perform better as compared to other hybrids in proline devoid media. Additionally, there might be threshold limit of proline, which helps in rapid green shoot regeneration in all the genotypes with the exception of IR20 x Mahulata.

The completion of androgenesis is marked by the root induction stage of AC. MS based media with increased auxin to cytokinin ratio is preferential for root induction. Increasing the sucrose concentration from 30 gm/l to 50 gm/l enable, the regenerants to achieve 100% root induction. Though it has been observed that shoot induction in callus requires low sugar concentration, the increase concentration of sucrose during the root induction might be due to the mass of shoot culture load.

The morpho-agronomic character of rice plant can easily depict its ploidy status. Often a haploid rice plant has diminutive stature and infertile tillers, clearly the plant looked like underdeveloped version of a normal plant. On contrary, the poly-ploids have gigantic stature with thick broad leaves and much more tiller count; these plants are also infertile [34]. However, rests of the fertile plants were considered as putative DHs until the identification of true DHs through molecular markers. Though a number of methods such as flow-cytometry [57] and cell-cytology are available to assess the ploidy level, STMS marker showed its potentiality in discrimination of diploids and DHs [29, 39].

## Abbreviations

DH: Doubled haploid
2,4-D: 2,4-Dichlorophenoxyacetic acid
BAP: 6-Benzylaminopurine
NAA: 1-Naphthaleneacetic acid
Kn: Kinetin
MS: Murashige and Skoog medium
AC: Anther culture
